# Risk Assessment and Management of Venous Thromboembolism in Women during Pregnancy and Puerperium (SAVE): An International, Cross-sectional Study

**DOI:** 10.1055/s-0038-1635573

**Published:** 2018-04-04

**Authors:** Jean-Christophe Gris, Joseph Aoun, Leyla Rzaguliyeva, Rowshan Begum, Hassan Salah, Tatia Tugushi, Mohammed Ghani-Chabouk, Mazen Zibdeh, Waleed Al Jassar, Joe Abboud, Nadia Meziane, Godwin-Olufemi Ajayi, Nazli Hossain, Alexey Pyregov, Hassan Abduljabbar, Leon C. Snyman, Radhouane Rachdi, Muna-Abdulrazzaq Tahlak, Dilbar Najmutdinova, the SAVE Study Group

**Affiliations:** 1 Department of Haematology, University of Montpellier and University Hospital of Nîmes, France; 2 Sanofi International Region, Antony, France; 3 Republican Clinical Hospital, Baku, Azerbaijan; 4 Holy Family Red Crescent Medical College and Hospital, Dhaka, Bangladesh; 5 Department of Gynecology and Obstetrics, Assiut University, Assiut, Egypt; 6 Reproductive Health Center “Fertimed,” Tbilisi, Georgia; 7 Salman Faeq Center, Baghdad, Iraq; 8 Department of Obstetrics and Gynaecology, Gardens Hospital, Amman, Jordan; 9 Maternity Hospital, Kuwait, Kuwait; 10 Hotel Dieu de France Hospital, Beirut, Lebanon; 11 Oum Albanine Clinic, Casablanca, Morocco; 12 Department of Obstetrics and Gynaecology, Lagos University Teaching Hospital, Lagos, Nigeria; 13 Department of Obstetrics and Gynecology, Dow University of Health Sciences, Karachi, Pakistan; 14 Scientific Center of Obstetrics, Gynecology and Perinatology, Moscow, Russia; 15 King Abdulaziz University Hospital, Jeddah, Kingdom of Saudi Arabia; 16 Department of Obstetrics and Gynaecology, University of Pretoria and Kalafong Provincial Tertiary Hospital, Pretoria, South Africa; 17 Gynecology and Obstetrics, Military Hospital, Tunis, Tunisia; 18 Department of Gynecology-Obstetrics, Latifa Hospital, Al Jaddaf, Dubai, United Arab Emirates; 19 Republican Specialized Scientific Practical Medical Center of Obstetrics and Gynecology, Tashkent, Uzbekistan

**Keywords:** pregnancy, prophylaxis, puerperium, risk assessment and management, venous thromboembolism

## Abstract

The clinical burden of obstetric venous thromboembolism (VTE) risk is inadequately established. This study assessed the prevalence and management of VTE risk during pregnancy and postpartum outside the Western world. This international, noninterventional study enrolled adult women with objectively confirmed pregnancy attending prenatal care/obstetric centers across 18 countries in Africa, Eurasia, Middle-East, and South Asia. Evaluations included proportions of at-risk women, prophylaxis as per international guidelines, prophylaxis type, factors determining prophylaxis, and physicians' awareness about VTE risk management guidelines and its impact on treatment decision. Data were analyzed globally and regionally. Physicians ( *N* = 181) screened 4,978 women, and 4,010 were eligible. Of these, 51.4% were at risk (Eurasia, 90%; South Asia, 19.9%), mostly mild in intensity; >90% received prophylaxis as per the guidelines (except South Asia, 77%). Women in Eurasia and South Asia received both pharmacological and mechanical prophylaxes (>55%), while pharmacological prophylaxis (>50%) predominated in Africa and the Middle-East. Low-molecular-weight heparin was the pharmacological agent of choice. Prophylaxis decision was influenced by ethnicity, assisted reproductive techniques, caesarean section, and persistent moderate/high titer of anticardiolipin antibodies, though variable across regions. Prophylaxis decision in at-risk women was similar, irrespective of physicians' awareness of guidelines (except South Asia). A majority (>80%) of the physicians claimed to follow the guidelines. More than 50% of women during pregnancy and postpartum were at risk of VTE, and >90% received prophylaxis as per the guidelines. Physicians are generally aware of VTE risk and comply with guidelines while prescribing prophylaxis, although regional variations necessitate efforts to improve implementation of the guidelines.

## Introduction

Obstetric venous thromboembolism (VTE) is a leading cause of maternal morbidity and mortality in the developed world. 1 2 Pregnant women are at a two- to 5fold higher risk of VTE versus nonpregnant women, 3 with an incidence of ∼1.2 to 1.6 per 1,000 deliveries. 4 5 Importantly, the risk is 60-fold higher in women during puerperium than in nonpregnant women. 3 The risk peaks at 3 to 6 weeks postpartum and declines rapidly thereafter, with a residual risk persisting up to 12 weeks postpartum. 6

The incidence of VTE has increased in the past decades. 7 VTE can be prevented by careful assessment of pre-existing and new-onset/transient risk factors, and employing optimum thromboprophylaxis. 7 Among multiple coexisting risk factors in women who develop VTE during pregnancy, previous pregnancy-linked VTE event is shown to be strongly correlated. 8 Other precipitating factors for VTE include an increased maternal age at delivery, use of assisted reproductive techniques (ART) for conception, 9 10 caesarean deliveries, 11 preeclampsia, obesity, immobility, thrombophilia, lupus, vascular disorders, and postpartum infection. 12 13

Around the world, more than two-thirds of the pregnant women have identifiable risk factors, yet the clinical burden of obstetric VTE in countries outside the Western world remains largely unknown. 5 Therefore, this study aimed at assessing the rate of women at VTE risk during pregnancy and puerperium in countries outside the Western world, that is, in the regions of Africa, Eurasia, the Middle-East, and South Asia. The study also determined the proportion of at-risk women who received prophylaxis as per the American College of Chest Physicians (ACCP) and/or Royal College of Obstetricians and Gynecologists (RCOG) guidelines, 8 14 prophylaxis type, factors driving physicians' prophylaxis decision, knowledge and proportion of physicians complying to international guidelines, and their attitude toward VTE prophylaxis.

## Materials and Methods

### Study Design and Setting

This international, noninterventional on the therapeutic strategy, cross-sectional study was conducted in 18 countries across Africa (Egypt, Morocco, Nigeria, Tunisia, and South Africa), Eurasia (Azerbaijan, Georgia, Russian Federation, and Uzbekistan), the Middle-East (Iraq, Jordan, Kuwait, Lebanon, Saudi Arabia, and United Arab Emirates), and South Asia (Bangladesh, India, and Pakistan) from December 2014 to October 2015.

### Physicians and Participants

Physicians were randomly contacted from a list of private and public practitioners provided by each participating country, and interested physicians were invited to participate.

Pregnant women (>18 years) with objectively confirmed pregnancy, visiting for the first prenatal consultation or any other consultation during pregnancy, with an existing medical condition, with/without the need for hospitalization, or labor/delivery care, and willingness to participate were enrolled. Women with a VTE event in the preceding 4 months or those using concurrent antithrombotic therapy for other medical reasons were excluded. Each site could enroll up to 30 women.

### Assessments

Assessments were made based on investigators' questionnaires and case report forms. Monitoring of source documents (quality control QC) was coordinated by a CRO (Altizem, Boulogne-Billancourt, France) on a random selection of sites defined through the list prepared by the Clinical Trial Operation Manager, per country. The total number of participating sites which were quality controlled was 10% of active sites at country level with a minimum of one site per country—site QC being systematically performed for the highest recruiting sites or for additional sites for which a QC was deemed necessary (e.g., poor quality of data collected, unexpected inclusion rates). It started after inclusion of 30% of patients at country level, and as soon as a site had included five patients.

The data recorded for pregnant and postpartum women included the timing of consultation (first trimester, weeks 1–13; second trimester, weeks 14–28; third trimester, week 29 to delivery; postpartum, 6 weeks postdelivery), demographics, vital signs and baseline characteristics, medical and obstetric history, family history of VTE, reasons for consultation/hospitalization, current treatment/medication, and risk assessment of VTE.

The investigators had been previously informed that the identification of patients at risk of VTE should be based on the available latest criteria published by the ACCP 8 or by the RCOG 14 (the latter transmitted before publication by Prof. C. Nelson-Piercy, to whom we send special thanks for her kind help). As we were very conscious of being unable to choose between any of these expert recommendations, we left the choice to each of the investigators to choose, in individual conscience, its reference system, and to stick to it. This choice was not a choice imposed within each country, but the choice of each investigator, exactly as in the current medical exercise. However, we reminded each medical coordinator in each country, before the start of the study, of the risk factors for venous thromboembolic disease of pregnancy as they appeared after a review of the available literature. This reminder was distributed to each investigator. The content of this reminder is available in the Supplementary Material .

The social and education status of the women included in the study was not systematically investigated or tested, and was not recorded.

The data recorded for physicians included demographics, location, specialty and years of practice, awareness of guideline(s), VTE risk assessment, and its management.

Appropriate prophylaxis against VTE was defined as corresponding to the ACCP 8 or to the RCOG 14 recommendations, without taking sides with either approach. Low-dose low-molecular-weight heparin (LMWH) prophylactic regimens corresponded to enoxaparin 40 mg daily, dalteparin 5,000 units daily, or tinzaparin 4,500 units daily, with no weight adjustment of doses. Intermediate-dose LMWH prophylactic regimen corresponded to the same doses injected twice daily, 12 hourly. Weight-adjusted dose LMWH regimens corresponded to enoxaparin 1 mg/kg twice daily, dalteparin 100 units/kg twice daily, or tinzaparin 175 units/kg daily.

### Statistical Analysis

Considering the qualitative nature of analysis, the sample size was calculated to ensure sufficient precision in the assessment of proportions. A precision from ± 9.8% to ± 2.1% could be obtained for assessing a percentage of 5 to 50, depending on the number of at-risk women per country and on the proportions assessed. An enrolment of 200 women per country was expected, and considering that data would not be available for 10% women, a total of 220 women per country were enrolled.

Quantitative data were summarized and presented as mean, standard deviation, median, range, and quartiles, while qualitative data were presented as number and percentage. The proportion of women at VTE risk, at-risk women receiving prophylaxis according to the ACCP/RCOG guidelines, and physicians following these guidelines were determined using the Clopper–Pearson method (two-sided 95% confidence interval [CI]). Factors were analyzed using univariate analysis. Chi-square or Fisher's exact test was used to assess the impact of each factor on VTE prophylaxis decision. Univariate odds ratios were presented with 95% CI.

### Ethics

The study was conducted as per the principles of the 18th World Medical Assembly (Helsinki 1964), guidelines for Good Epidemiology Practice (US15 and European16), and was compliant with all international guidelines and national laws of the participating countries. The protocol was approved by the local Institutional Review Board/Independent Ethics Committee of each country, and regulatory submissions were performed in accordance with local data protection regulations. All participating women signed a written informed consent before starting the study.

## Results

### Physician and Participant Disposition and Characteristics

A total of 181 physicians participated in the study, mostly from urban areas, practicing on an average for more than 20 years as both obstetricians and gynecologists. The Middle-East had a parity of male and female physicians, while in other regions, either male or female physicians were predominant. Public health centers were the main work places in Eurasia and Africa, whereas private medical support was predominant in the Middle-East. South Asia had a predominance of both public and private setups. Weekly consultations during pregnancy were maximum in South Asia and minimum in Eurasia ( Table 1 ).

**Table 1:** Physician demographics and characteristics

Characteristics	Global ( *N* = 181)	Africa ( *N* = 39)	Eurasia ( *N* = 49)	Middle-East ( *N* = 50)	South Asia ( *N* = 43)
Age, y (mean ± SD)	50.5 ± 8.6	52.5 ± 9.3	48.8 ± 8.0	50.0 ± 7.8	51.1 ± 9.4
Gender, *n* (%)					
Male	74 (40.9)	32 (82.1)	10 (20.4)	25 (50.0)	7 (16.3)
Female	107 (59.1)	7 (17.9)	39 (79.6)	25 (50.0)	36 (83.7)
Years of practice (mean ± SD)	22.6 ± 8.6	23.6 ± 9.0	23.4 ± 7.7	22.0 ± 7.8	21.5 ± 10.0
Main work place, %					
Public	32.0	46.2	61.2	16.0	4.7
Private	34.8	12.8	26.5	54.0	41.9
Both	33.1	41.0	12.2	30.0	53.5
Location, %					
Urban	96.7	100	93.9	98.0	95.3
Rural	3.3	0	6.1	2.0	4.7
Pregnant women consulted/wk (mean ± SD)	80.9 ± 79.3 b	94.4 ± 83.7	38.7 ± 26.9	85.5 ± 63.5 b	111.6 ± 108.5
Specialty, % a					
Obstetrician	13.3	10.3	14.3	18.0	9.3
Gynecologist	14.9	5.1	38.8	4.0	9.3
Obstetrician + Gynecologist	63.5	76.9	34.7	68.0	79.1

Abbreviation: SD, standard deviation.

Notes: Africa comprises Egypt, Morocco, Nigeria, Tunisia, and South Africa; Eurasia comprises Azerbaijan, Georgia, Russian Federation, and Uzbekistan; the Middle-East comprises United Arab Emirates, Iraq, Jordan, Kuwait, Lebanon, and Saudi Arabia; South Asia comprises Bangladesh, India, and Pakistan.

a Remaining physicians belonged to the specialties: other, obstetrician + other, gynecologist + other, obstetrician + gynecologist + other.

b Data of one physician was missing.

**Fig. 1:**
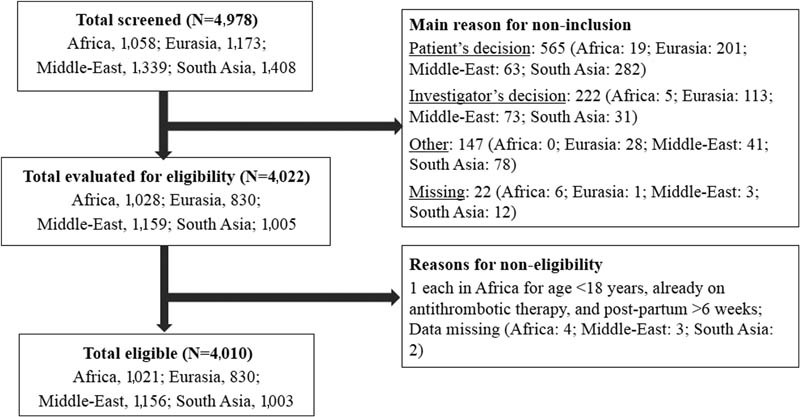
Participant disposition. Africa comprises Egypt, Morocco, Nigeria, Tunisia, and South Africa; Eurasia comprises Azerbaijan, Georgia, Russian Federation, and Uzbekistan; the Middle-East comprises United Arab Emirates, Iraq, Jordan, Kuwait, Lebanon, and Saudi Arabia; South Asia comprises Bangladesh, India, and Pakistan.

Of the 4,978 women screened globally, 4,010 women participated in the study ( Fig. 1 ). The mean age of enrolled women was 29.4 years. The majority of women were Caucasian, except for those in South Asia. Obesity (body mass index [BMI] >30 kg/m ^2^ ) was most prevalent in Africa and least in Eurasia. However, Eurasia witnessed the highest prevalence of VTE risk factors like smoking; gross varicose veins; previous abnormal pregnancies; previous superficial vein thrombosis; and family histories of VTE, cancer, and thrombophilia. Venous insufficiency was predominantly mild (as per the Clinical-Etiology-Anatomy-Pathophysiology classification system) 15 globally, and was again most prevalent in Eurasia. On the basis of these risk factors at baseline, South Asian women were perceived to be at the lowest risk of VTE ( Table 2 ). Majority of the eligible women received consultation during pregnancy (90.1%), particularly during the last trimester (38.1%), and were enrolled mainly during their routine visit to the physician (81.9%). Consultation visits during the postpartum period were the lowest in South Asia (7.2%) and the highest in Africa (12.7%; Table 3 ).

**Table 2:** Participant demographics and characteristics

Characteristics	Global ( *N* = 4,010)	Africa ( *N* = 1,021)	Eurasia ( *N* = 830)	Middle-East ( *N* = 1,156)	South Asia ( *N* = 1,003)
Age, y (mean ± SD)	29.4 ± 5.4	30.2 ± 5.5	29.5 ± 5.8	29.9 ± 5.4	27.9 ± 4.9
Ethnicity, %					
Caucasian	55.5	58.2	80.4	83.4	<0.1
Black	10.4	38.8	0.0	1.8	0.0
South Asian	30.2	2.4	4.3	14.7	97.9
Other	3.8	0.6	15.3	<0.1	2.0
BMI					
Mean ± SD, kg/m ^2^	27.4 ± 5.7	29.2 ± 6.3	25.5 ± 4.7	28.1 ± 5.9	26.5 ± 5.0
>30 kg/m ^2^ , %	26.5	37.7	14.8	29.3	21.7
Smokers, %	5.3	3.1	12.2	6.2	0.9
Gross varicose veins, %	8.2	12.2	13.9	6.7	1.0
Venous insufficiency, a %					
Mild disease	9.3	8.3	27.1	4.6	0.9
Intermediate disease	2.9	2.0	7.8	1.9	1.0
Severe disease	0.1	0.0	0.6	0.0	0.0
Number of previous pregnancies (mean ± SD)	1.4 ± 1.6	1.5 ± 1.4	1.6 ± 1.6	1.7 ± 1.8	1.0 ± 1.3
Previous abnormal pregnancies, %	31.3	26.0	48.4	30.7	23.1
Previous VTE, %	1.6	2.4	1.3	2.2	0.4
Previous superficial vein thrombosis, %	1.6	1.1	4.7	1.1	0.2
Previous arterial ischemic events, %	0.2	0.3	0.2	<0.1	<0.1
Family history, %					
VTE	5.8	3.4	14.8	5.4	1.0
Cancer known	4.2	4.2	5.9	4.2	2.8
Thrombophilia screening: yes	1.4	0.4	5.8	0.3	<0.1
Thrombophilia screening: unknown	21.4	7.3	33.1	24.3	22.7

Abbreviations: BMI, body mass index; SD, standard deviation; VTE, venous thromboembolism.

Notes: Africa comprises Egypt, Morocco, Nigeria, Tunisia, and South Africa; Eurasia comprises Azerbaijan, Georgia, Russian Federation, and Uzbekistan; the Middle-East comprises United Arab Emirates, Iraq, Jordan, Kuwait, Lebanon, and Saudi Arabia; South Asia comprises Bangladesh, India, and Pakistan.

a Defined as per the clinical classification of Clinical-Etiology-Anatomy-Pathophysiology (CEAP) for chronic venous disorders (Eklöf et al 15 ); mild disease: C1 or C2 original levels; intermediate: C3 or C4 levels; severe: C5 or C6 levels.

**Table 3:** Timing of consultation and reasons for consultation/hospitalization

	Global ( *N* = 4,010)	Africa ( *N* = 1,021)	Eurasia ( *N* = 830)	Middle-East ( *N* = 1,156)	South Asia ( *N* = 1,003)
Timing of consultation, *n* (%)					
During pregnancy	3,614 (90.1)	891 (87.3)	751 (90.5)	1,041 (90.1)	931 (92.8)
During postpartum	396 (9.9)	130 (12.7)	79 (9.5)	115 (9.9)	72 (7.2)
Timing, *n* (%)					
1st trimester	900 (22.4)	142 (13.9)	276 (33.3)	276 (23.9)	206 (20.5)
2nd trimester	1,188 (29.6)	289 (28.3)	278 (33.5)	312 (27.0)	309 (30.8)
3rd trimester	1,526 (38.1)	460 (45.1)	197 (23.7)	453 (39.2)	416 (41.5)
1–2 wk postpartum	348 (8.7)	119 (11.7)	71 (8.6)	90 (7.8)	68 (6.8)
2–4 wk postpartum	34 (0.8)	5 (0.5)	6 (0.7)	19 (1.6)	4 (0.4)
> 4 wk postpartum	14 (0.3)	6 (0.6)	2 (0.2)	6 (0.5)	0
For pregnancy: Gestational week of consultation/hospitalization					
Number	3,613	890	751	1,041	931
Median (range)	25 (2–42)	29 (2–42)	19 (4–41)	26 (3–42)	27 (4–41)
For puerperium: Week of consultation/hospitalization					
Number	395	130	79	115	71
Median (range)	1 (1–6)	1 (1–5)	1 (1–5)	1 (1–6)	1 (1–3)
Reasons for consultation, *n* (%)					
Routine visit	3,282 (81.9)	767 (75.2)	615 (74.1)	1,023 (88.5)	877 (87.4)
Gestational anemia	517 (12.9)	59 (5.8)	309 (37.2)	77 (6.7)	72 (7.2)
Caesarean delivery	465 (11.6)	190 (18.6)	115 (13.9)	101 (8.7)	59 (5.9)
Preexisting chronic hypertension, hypertension in pregnancy	255 (6.4)	75 (7.3)	81 (9.8)	42 (3.6)	57 (5.7)
Gestational diabetes mellitus	226 (5.6)	87 (8.5)	35 (4.2)	53 (4.6)	51 (5.1)
Placenta-mediated pregnancy complication	196 (4.9)	69 (6.8)	65 (7.8)	27 (2.3)	35 (3.5)
Vaginal delivery	179 (4.5)	68 (6.7)	27 (3.3)	58 (5.0)	26 (2.6)
Intercurrent disease potentially necessitating surgery/hospitalization	129 (3.2)	60 (5.9)	8 (1.0)	38 (3.3)	23 (2.3)
Intercurrent infection	120 (3.0)	21 (2.1)	55 (6.6)	22 (1.9)	22 (2.2)
Preeclampsia	122 (3.0)	52 (5.1)	34 (4.1)	11 (1.0)	25 (2.5)
Gestational thrombocytopenia	110 (2.7)	19 (1.9)	79 (9.5)	8 (0.7)	4 (0.4)
Intrauterine growth restriction	79 (2.0)	25 (2.4)	21 (2.5)	16 (1.4)	17 (1.7)
Pregnancy loss	82 (2.0)	35 (3.4)	4 (0.5)	27 (2.3)	16 (1.6)
Intercurrent renal function impairment	59 (1.5)	9 (0.9)	47 (5.7)	3 (0.3)	0
Premature rupture of the membranes	59 (1.5)	21 (2.1)	11 (1.3)	16 (1.4)	11 (1.1)
Maternal constitutional hemoglobin disease	45 (1.1)	6 (0.6)	33 (4.0)	3 (0.3)	3 (0.3)
Placental abruption	40 (1.0)	13 (1.3)	17 (2.0)	7 (0.6)	3 (0.3)
Fetal distress	39 (1.0)	20 (2.0)	2 (0.2)	11 (1.0)	6 (0.6)
Surgical procedure (e.g., appendicectomy), pregnancy, or puerperium	36 (0.9)	11 (1.1)	4 (0.5)	14 (1.2)	7 (0.7)
Intercurrent jaundice/liver or biliary tract disease	33 (0.8)	5 (0.5)	21 (2.5)	3 (0.3)	4 (0.4)
Blood transfusion for postpartum hemorrhage	32 (0.8)	18 (1.8)	3 (0.4)	8 (0.7)	3 (0.3)
Intercurrent inflammatory disease outbreak	30 (0.7)	4 (0.4)	23 (2.8)	1 (<0.1)	2 (0.2)
Maternal-fetal alloimmunization	29 (0.7)	6 (0.6)	18 (2.2)	3 (0.3)	2 (0.2)
Intercurrent trauma	21 (0.5)	10 (1.0)	3 (0.4)	7 (0.6)	1 (<0.1)
Caesarean infection	20 (0.5)	7 (0.7)	0	11 (1.0)	2 (0.2)
Postpartum wound infection	17 (0.4)	9 (0.9)	1 (0.1)	4 (0.3)	3 (0.3)

Notes: Africa comprises Egypt, Morocco, Nigeria, Tunisia, and South Africa; Eurasia comprises Azerbaijan, Georgia, Russian Federation, and Uzbekistan; the Middle-East comprises United Arab Emirates, Iraq, Jordan, Kuwait, Lebanon, and Saudi Arabia; South Asia comprises Bangladesh, India, and Pakistan.

### Proportion and Intensity of VTE Risk

**Fig. 2:**
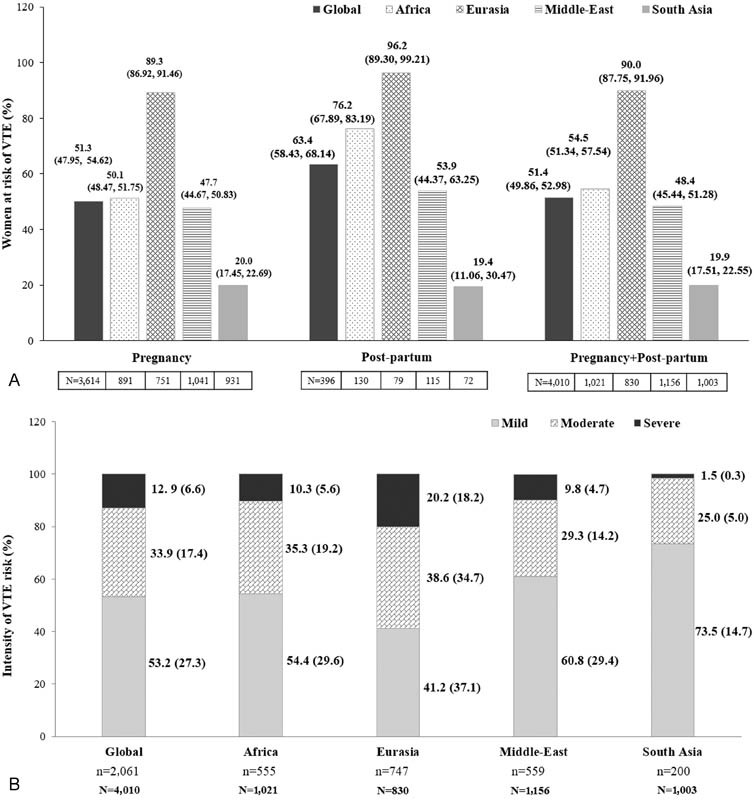
( **A** ) Proportion and ( **B** ) intensity of VTE risk. VTE, venous thromboembolism; “ *n* ” represents the total number of women for whom data were collected. “ *N* ” represents the total eligible population. ( **A** ) Values are presented as % participants (95% confidence interval). ( **B** ) The bold text indicates the percentage of at-risk women calculated from the total eligible population ( *N* ). Africa comprises Egypt, Morocco, Nigeria, Tunisia, and South Africa; Eurasia comprises Azerbaijan, Georgia, Russian Federation, and Uzbekistan; the Middle-East comprises United Arab Emirates, Iraq, Jordan, Kuwait, Lebanon, and Saudi Arabia; South Asia comprises Bangladesh, India, and Pakistan.

Globally, half (51.4%) of the participating women were at VTE risk during pregnancy and postpartum, with the highest risk in Eurasia (90%) and the lowest in South Asia (19.9%). A similar trend was seen in the subpopulation of pregnant women, while the risk was higher during the postpartum period than during pregnancy ( Fig. 2A ). The risk was mostly mild across regions; however, Eurasia had the highest proportion of high-risk women (20.2 vs. 1.5% in South Asia and 12.9% globally; Fig. 2B ).

### Venous Thromboembolism Prophylaxis

#### Rate and Period of Prophylaxis

**Fig. 3:**
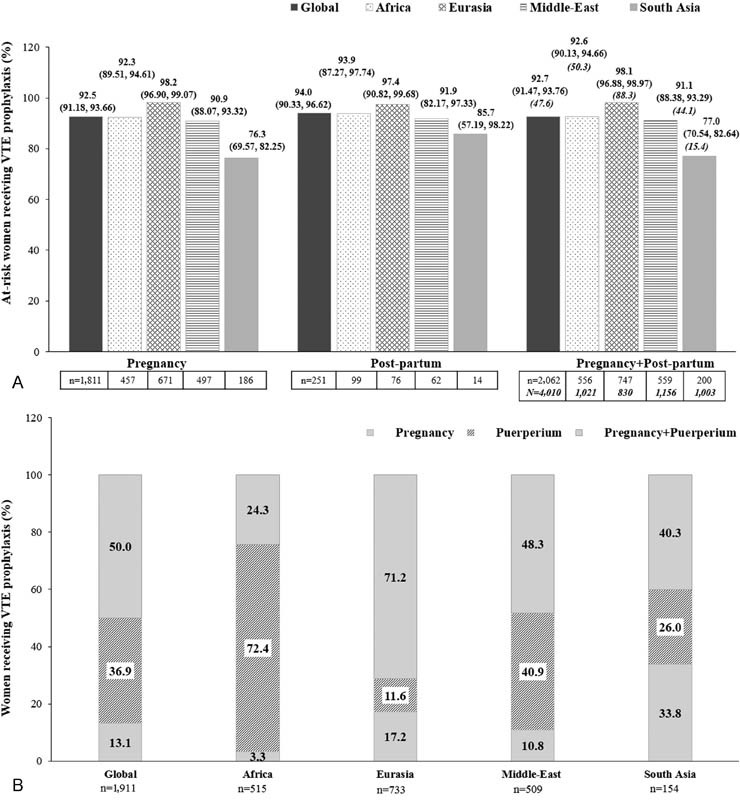
( **A** ) Women at risk of VTE receiving prophylaxis according to the ACCP and/or RCOG guidelines and ( **B** ) the period of prescribing prophylaxis. VTE, venous thromboembolism; “ *n* ” represents the total number of women at risk of VTE, for whom data were collected. “ *N* ” represents the total eligible population. ( **A** ) Values are presented as % participants (95% confidence interval); the bold italic text indicates the percentage of at-risk women receiving prophylaxis, calculated from the total eligible population ( *N* ). Africa comprises Egypt, Morocco, Nigeria, Tunisia, and South Africa; Eurasia comprises Azerbaijan, Georgia, Russian Federation, and Uzbekistan; the Middle-East comprises United Arab Emirates, Iraq, Jordan, Kuwait, Lebanon, and Saudi Arabia; South Asia comprises Bangladesh, India, and Pakistan.

The majority of at-risk women received prophylaxis consistently across the regions, except South Asia, wherein nearly 23% of the women did not receive prophylaxis as per the ACCP/RCOG guidelines with a similar trend in pregnant and postpartum women ( Fig. 3A ).

Globally, VTE prophylaxis was prescribed to nearly half of the medically examined women (47.7%): most frequently in Eurasia (88.3%), less frequently in Africa (50.4%) and the Middle-East (44.0%), and rarely in South Asia (15.4%). Half of the women enrolled in the study received prophylaxis, both during pregnancy and puerperium, while 13.1 and 36.9% received prophylaxis exclusively during pregnancy and puerperium, respectively. Prophylaxis exclusively during puerperium was the lowest in Eurasia (11.6%) and the highest in Africa (72.4%; Fig. 3B ).

#### Type of Prophylaxis

**Fig. 4:**
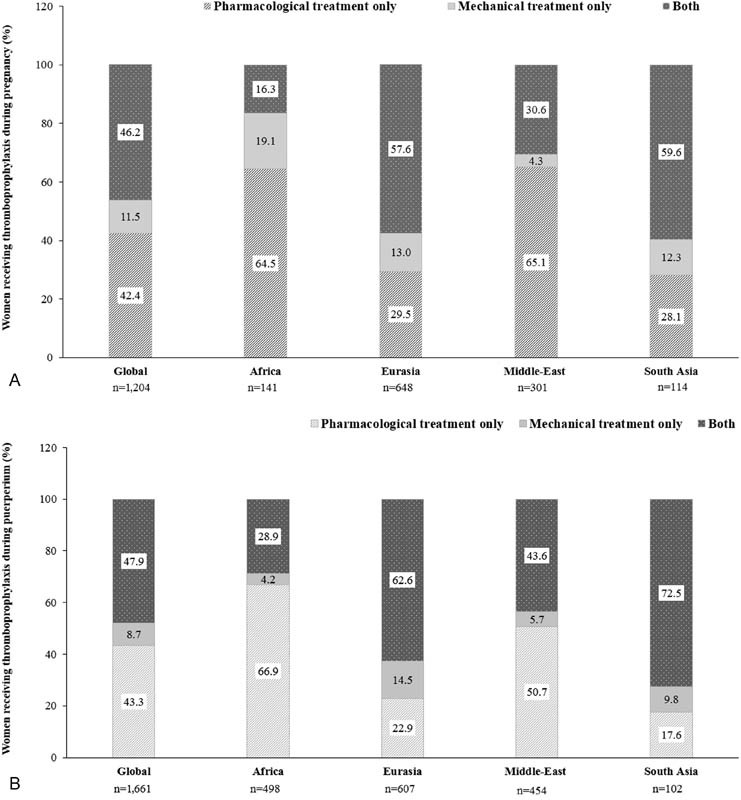
Type of thromboprophylaxis during ( **A** ) pregnancy and ( **B** ) puerperium. “ *n* ” represents the total number of women for whom data were collected. Africa comprises Egypt, Morocco, Nigeria, Tunisia, and South Africa; Eurasia comprises Azerbaijan, Georgia, Russian Federation, and Uzbekistan; the Middle-East comprises United Arab Emirates, Iraq, Jordan, Kuwait, Lebanon, and Saudi Arabia; South Asia comprises Bangladesh, India, and Pakistan.

Overall, 42.4% received pharmacological, 11.5% received mechanical, and 46.2% received both types of prophylaxes. In Eurasia and South Asia, >55% women received both prophylaxes; however, pharmacological treatment was predominant in Africa and the Middle-East ( Fig. 4A ). The pattern of thromboprophylaxis prescribed during pregnancy was the same as that during puerperium ( Fig. 4B ). Globally, 78.6% (834/1,061) women received pharmacological prophylaxis from the 20.3 ± 10.5 weeks until delivery (median: 20th gestational week with interquartile [Q1:Q3] interval [12.0:30.0 weeks]), with 12.6% of women receiving it during the second and third trimesters and 4.1% during the third trimester only. Globally, 75.3% (1,138/1,511) of women received pharmacological prophylaxis starting before the second day of delivery (median of 1.0 with Q1:Q3 interval [1.0:1.0 weeks]) until 2 to 6 weeks postpartum (36.5 and 13.1%, respectively) or until 5 to 10 days post–caesarean delivery (25.7%).

Globally, LMWH was more frequently prescribed than aspirin during pregnancy (1.6-fold), and a similar trend was noted in Africa, the Middle-East, and Eurasia (1.2-fold, 1.4-fold, and 2.3-fold, respectively), except in South Asia, where aspirin was more frequently prescribed than LMWH during pregnancy (2.0-fold). Similarly, LMWH was more frequently prescribed than aspirin during puerperium globally (4.4-fold), and also across the Middle-East (4.4-fold), South Asia (3.4-fold), and Eurasia (2.6-fold), while in Africa, it was 14.7-fold. The brands of LMWH prescribed to the women were not recorded. The used LMWH doses were the following: during pregnancy—low doses in 54.5%, intermediate doses in 22.9%, and weight-adjusted doses in 22.6%; during puerperium—low doses in 57.9%, intermediate doses in 22.6%, and weight-adjusted doses in 19.5%.

Globally, aspirin was more frequently prescribed during pregnancy than during puerperium (2.4-fold), while LMWH was equally prescribed during both pregnancy and puerperium ( Table 4 ). The reasons for low-dose aspirin treatments were not specifically questioned and recorded. Globally, aspirin was prescribed in pregnant women with at least one abnormal previous pregnancy (any previous placenta-mediated complication [PMC] or pregnancy loss) in 56.2% of the cases, in absence of any abnormal previous pregnancy in 44.8% of the cases. Only 15.3% of the women who received aspirin had a PMC history. Aspirin was also significantly prescribed during puerperium. As the antithrombotic properties of aspirin, even if not previously investigated and tested during pregnancy and puerperium, are currently evoked, this led us to present aspirin uses as part of the antithrombotic strategies used by the managing physicians. However, we must recognize that these practices are very different from expert recommendations, and constitute a significant limitation to the general acceptability of prescribing antithrombotic prophylaxis during pregnancy and postpartum.

**Table 4:** Type of pharmacological treatment a

	Global ( *N* = 4,010)		Africa ( *N* = 1,021)		Eurasia ( *N* = 830)		Middle-East ( *N* = 1,156)		South Asia ( *N* = 1,003)	
	Pregnancy	Puerperium	Pregnancy	Puerperium	Pregnancy	Puerperium	Pregnancy	Puerperium	Pregnancy	Puerperium
Number	1,066	1,515	114	477	564	519	288	427	100	92
Low-molecular-weight heparin	783 (73.5)	1,283 (84.7)	75 (65.8)	429 (89.9)	447 (79.3)	400 (77.1)	224 (77.8)	379 (88.8)	37 (37.0)	75 (81.5)
Aspirin	494 (46.3)	289 (19.1)	64 (56.1)	29 (6.1)	197 (34.9)	151 (29.1)	159 (55.2)	87 (20.4)	74 (74.0)	22 (23.9)
Unfractionated heparin	11 (1.0)	52 (3.4)	2 (1.8)	24 (5.0)	5 (0.9)	2 (0.4)	4 (1.4)	24 (5.6)	0	2 (2.2)
New oral anticoagulants	12 (1.1)	40 (2.6)	0	8 (1.7)	12 (2.1)	14 (2.7)	0	17 (4.0)	0	1 (1.1)
Other	32 (3.0)	17 (1.1)	0	1 (0.2)	29 (5.1)	15 (2.9)	2 (0.7)	0	1 (1.0)	1 (1.1)
Missing	0	1	0	0	0	0	0	1	0	0

Notes: Values are presented as *n* (%). Africa comprises Egypt, Morocco, Nigeria, Tunisia, and South Africa; Eurasia comprises Azerbaijan, Georgia, Russian Federation, and Uzbekistan; the Middle-East comprises United Arab Emirates, Iraq, Jordan, Kuwait, Lebanon, and Saudi Arabia; South Asia comprises Bangladesh, India, and Pakistan.

a May include several types of pharmacological treatments.

One of the underlying goals of this observational study was also to evaluate off-label use of drugs, with special attention for the most recent and critical ones in that field, without investigating the reasons which led physicians to do so. This is why we specifically observed for any direct oral anticoagulants prescription during puerperium ( *N* = 40: in the four continent/subcontinents) and also during pregnancy ( *N* = 12: only in Eurasia). Doses and brands were not recorded.

#### Risk Factors Determining Prophylaxis

Globally, ethnicity (significantly lower in black and South Asian women than in Caucasian, both *p* < 0.0001), use of ART *(p* = 0.0106), caesarean delivery ( *p* < 0.0001), and persistent presence of moderate/high titer of anticardiolipin antibodies ( *p* = 0.0202; not systematically monitored: only screened in the women with a personal history which may have evoked an antiphospholipid syndrome) seemed to significantly influence the prophylaxis prescription versus other women.

However, there were variations across regions, and no specific factor was identified in Eurasia ( Table 5 ).

**Table 5:** Factors determining VTE prophylaxis

		Odds ratio (95% CI)	*p* -Value
Global			
Assisted reproductive technique		4.51 (1.42, 14.30)	0.0106
Caesarean delivery		4.45 (2.17, 9.15)	<0.0001
Ethnicity	Black a	0.27 (0.17, 0.43)	<0.0001
	South Asian a	0.22 (0.15, 0.33)	<0.0001
Persistent moderate/high titer anticardiolipin antibodies		10.43 (1.44, 75.51)	0.0202
Africa			
BMI >30 kg/m ^2^		0.52 (0.27, 0.99)	0.0467
Ethnicity	Black a	0.21 (0.11, 0.43)	<0.0001
Caesarean section		6.46 (1.97, 21.23)	0.0021
Eurasia (no specific reasons identified)			
Middle-East			
BMI ≥ 25 kg/m ^2^		1.98 (1.08, 3.63)	0.0276
Caesarean section		4.34 (1.03, 18.24)	0.0448
South Asia			
Immobility		0.14 (0.04, 0.51)	0.0029

Abbreviations: BMI, body mass index; CI, confidence interval; VTE, venous thromboembolism.

Notes: Africa comprises Egypt, Morocco, Nigeria, Tunisia, and South Africa; Eurasia comprises Azerbaijan, Georgia, Russian Federation, and Uzbekistan; the Middle-East comprises United Arab Emirates, Iraq, Jordan, Kuwait, Lebanon, and Saudi Arabia; South Asia comprises Bangladesh, India, and Pakistan.

a In comparison with Caucasians.

In Africa, prophylaxis was less frequently prescribed to black or overweight women (BMI >30 kg/m ^2^ ) with a perceived VTE risk than to Caucasians ( *p <* 0.0001) and the other women ( *p* = 0.0467), respectively, and more frequently to women undergoing caesarean delivery than to other women ( *p* = 0.0021).

In the Middle-East, prophylaxis prescribed to overweight women (BMI ≥25 kg/m ^2^ ) or women undergoing caesarean delivery was more frequent than that prescribed to other women ( *p* = 0.0276 and *p* = 0.0448, respectively).

In South Asia, prophylaxis was less frequently prescribed to women with reduced mobility than to other women ( *p* = 0.0029).

#### Reasons for Not Prescribing Prophylaxis

**Fig. 5:**
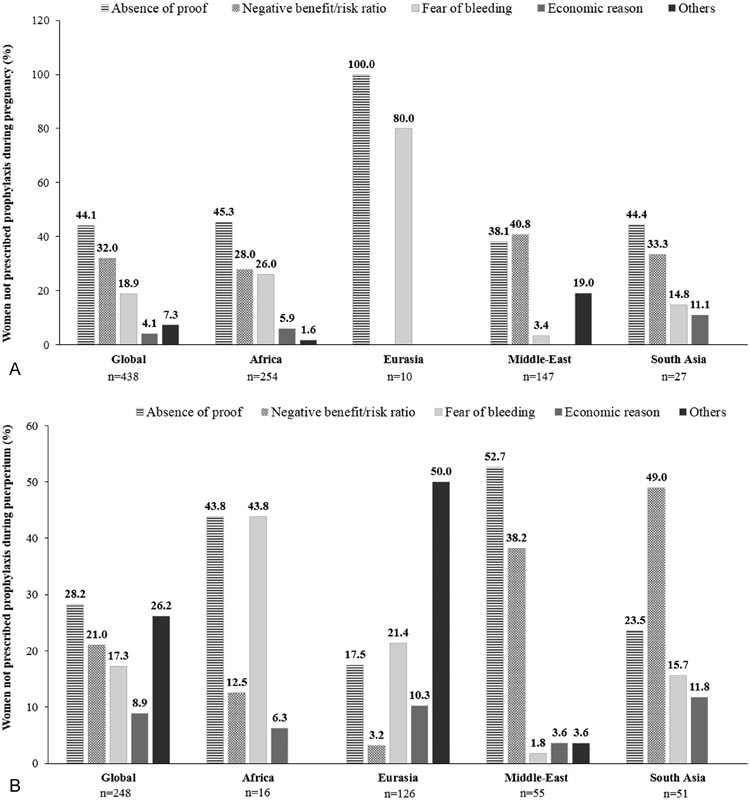
Reasons for not prescribing thromboprophylaxis during ( **A** ) pregnancy and ( **B** ) puerperium. “ *n* ” represents the total number of women for whom data were collected. *Note* : Several reasons can be listed for one patient. Africa comprises Egypt, Morocco, Nigeria, Tunisia, and South Africa; Eurasia comprises Azerbaijan, Georgia, Russian Federation, and Uzbekistan; the Middle-East comprises United Arab Emirates, Iraq, Jordan, Kuwait, Lebanon, and Saudi Arabia; South Asia comprises Bangladesh, India, and Pakistan.

Globally, only 7.3% of women at VTE risk during pregnancy and puerperium were not prescribed prophylaxis. The trend was nearly similar across regions, except in South Asia ( Fig. 3A ). The most frequent reasons for not prescribing prophylaxis during pregnancy were the absence of evidence of benefit (44.1%), negative benefit/risk ratio (32.0%), fear of bleeding (18.9%), and economic reasons (4.1%). Further, a similar trend in reasons was reported in Africa and South Asia; the perceived negative benefit/risk ratio (40.8%) was the main reason for not prescribing prophylaxis in the Middle-East, whereas only absence of proof (100%) and fear of bleeding (80%) were cited as key reasons in Eurasia ( Fig. 5A ). Similar reasons were reported during puerperium, although the percentages varied ( Fig. 5B ).

#### Physicians' Awareness and Adherence to International Guidelines

**Fig. 6:**
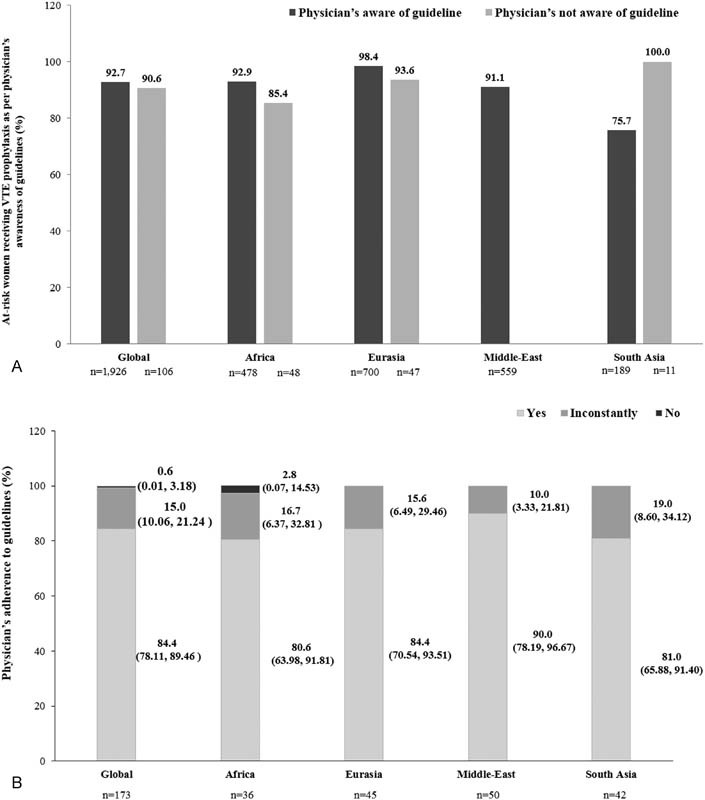
( **A** ) Prophylaxis prescription in women at risk of VTE as per physicians' awareness of guidelines and ( **B** ) physicians' adherence to guidelines. VTE, venous thromboembolism; “ *n* ” represents the total women for whom data were collected. ( **B** ) Values are presented as % physicians (95% confidence interval). Africa comprises Egypt, Morocco, Nigeria, Tunisia, and South Africa; Eurasia comprises Azerbaijan, Georgia, Russian Federation, and Uzbekistan; the Middle-East comprises United Arab Emirates, Iraq, Jordan, Kuwait, Lebanon, and Saudi Arabia; South Asia comprises Bangladesh, India, and Pakistan.

Irrespective of physicians' awareness regarding guidelines, globally, there was no difference in the prescription of VTE prophylaxis in at-risk women in different regions, except in South Asia. In South Asia, the prophylaxis prescriptions by physicians who were not aware of the guidelines were higher, although there were fewer physicians in this category. In the Middle-East, all physicians claimed to be aware of guidelines ( Fig. 6A ). More than 80% of physicians globally and in each region were reported to follow the guidelines, while in Africa, 2.8% of physicians declared that they did not follow the guidelines ( Fig. 6B ).

## Discussion

This multinational study conducted across 18 countries in over 4,000 women during pregnancy and puerperium provides key insights into the VTE risk assessment and management. To the best of our knowledge, this is the first observational study conducted globally and exclusively in this indication and population on such a large scale.

This study showed that globally, half (51.4%) of the women during pregnancy and postpartum were perceived by physicians to be at VTE risk, mostly mild in intensity. The risk was higher during the postpartum period, consistent with previous findings. 16 However, perceived VTE risk in the present study was higher than that reported in two previous cross-sectional studies. In a noninterventional, multinational study conducted at seven centers in the Arabian Gulf countries, 32% (1,337 of 4,131) of the eligible pregnant women were at VTE risk, 17 almost equal to two-thirds of that observed for the Middle-East in the present study. In another study in Ireland, 40% (145 of 364) of the pregnant and postpartum women were at risk, 18 nearly half of that observed for Eurasia in the present study. This notable difference in perceived risk could be attributed to a high incidence of previous abnormal pregnancies in the present study (31.3%), possibly impacting physicians' perception of risk. The perceived risk was highest in Eurasia (90%), both during pregnancy and postpartum. This could be partly attributed to the fact that VTE risk factors such as smoking, gross varicose veins, previous abnormal pregnancies, previous superficial vein thrombosis, venous insufficiency, family histories of VTE, cancer, and thrombophilia were most frequently reported in Eurasia. On the contrary, South Asia presented the lowest perceived risk, which could be partly attributed to less frequently reported risk factors in this region.

The increased VTE prophylaxis in the present study correlates with the perceived risk, with majority of at-risk women (>90%) receiving prophylaxis as per the guidelines. This trend appears clinically favorable, as timely and adequate prophylaxis reduces VTE-related morbidity and mortality because of a narrow therapeutic window. As expected, the frequency of prophylaxis in the present study was also much higher than that reported in the two previous cross-sectional studies in Arabian Gulf countries and Ireland (8.3 and 69% at-risk women receiving prophylaxis, respectively). 17 18 Interestingly, the data also showed that the proportion of at-risk women receiving prophylaxis was lower in South Asia, probably because of two reasons. First, a misconception that obstetric VTE incidence is lower in Asian women than in Caucasian women, thereby negatively impacting the prophylaxis decision. 19 Second, the BMI cut-off for obesity is lower for Asians (>27 kg/m ^2^ ) than for Caucasians (≥30 kg/m ^2^ ); however, this difference is apparently not considered while evaluating VTE risk in Asian women, and the cut-off for Caucasians is often applied to Asian women as well. 20 21

The study also showed that prophylaxis was more commonly prescribed during puerperium than during pregnancy, while about half of the at-risk women received prophylaxis during both pregnancy and puerperium. This trend was expected because VTE risk is greater during puerperium than during pregnancy. 3

Additionally, nearly half of the prophylaxis globally comprised a combination of both mechanical and pharmacological treatments during pregnancy. Eurasia and South Asia followed a similar trend, while pharmacological treatment was predominantly used in Africa and the Middle-East. Mechanical prophylaxis with elastic stockings or intermittent pneumatic compression can be used in pregnant women with high risk of VTE. 8 However, there is limited evidence to prove that these devices are effective. In case of pharmacological prophylaxis, though enormous evidence is available on its safety and efficacy, 22 majority of it is derived from case studies, expert opinion, and extrapolations from nonpregnant women, 23 thereby necessitating the need for randomized controlled trials.

In agreement with this evidence, recent clinical practice guidelines also recommend pharmacological prophylaxis, particularly LMWH, in women at VTE risk. 8 14 The LMWH prescription in the present study was in accordance with the ACCP and RCOG guidelines 8 14 and published data. 24 However, prescription of aspirin was not as per the guidelines and was preferred over LMWH in South Asia. A striking fact revealed by this study is that the vast majority of aspirin prescriptions during pregnancy and postpartum are not performed for its primary indication, the prevention of PMCs, but to cover venous thrombotic risk. This is a misuse that will have to be addressed.

Factors determining prophylaxis varied across regions. ART and caesarean delivery strongly determined prophylaxis prescription and were in line with results of earlier studies where VTE incidence was higher in pregnancies using ART and caesarean delivery. 11

The present study also provided insights into physicians' adherence to maternal evidence-based thromboprophylaxis guidelines, as there is lack of information, and the available data pertains to few nationalized cohort studies only. 18 25 Majority of the participating physicians across the studied countries claimed to follow international guidelines. Consequently, no difference could be drawn regarding their attitude to VTE prophylaxis, with respect to awareness of guidelines, except in South Asia. This could again be attributed to the general misconception of a lower VTE incidence in South Asia, and the lack of stringently defined and applied BMI cut-off in this region. In a study involving 400 obstetricians across the United States, 77.1% followed the American College of Obstetricians and Gynecologists guidelines, 38.2% followed local hospital guidelines, while 21.7 and 3.3% followed the ACCP guidelines and other international guidelines, respectively, at the time of caesarean delivery. 26

### Strengths and Limitations

Participant characteristics were homogeneous with respect to age, BMI, and the number of previous pregnancies. Similarly, physician characteristics were homogenous with respect to age and work experience. Therefore, the results can be considered representative of a real-world scenario, and can be extrapolated to a larger population.

The study also has few limitations. The African countries which were involved in this study represented one country of Central Africa (Nigeria), the populations of North Africa and South Africa being the majority. Therefore, we cannot claim to summarize the overall practice in Africa. More urban than rural sites participated, precluding the extrapolation of these data to rural health care centers where access to advanced health care is limited. Moreover, the rate of at-risk pregnant women might have been overestimated compared with the true global rate in this population, as physicians might have cautiously and diligently assessed VTE risk while aligning themselves to the study. Additionally, owing to the results and a very high percentage of at-risk women receiving a prescription of VTE prophylaxis, the multivariate analysis, which was considered in the statistical analysis plan, was not performed. Indeed, the data could not be analyzed in a validated regression model.

## Conclusion

In our study, a high proportion of women who received consultation during pregnancy and postpartum are at VTE risk, and a majority of them are being prescribed appropriate thromboprophylaxis in accordance with the international guidelines by the physicians. Consulting physicians are aware of the VTE risk during pregnancy and postpartum and are committed to identifying this risk in their patients and managing them. However, some differences and discrepancies are observed among the regions, necessitating efforts to further develop evidence-based guidelines and their implementation.

## Notes

Appendix: SAVE Study Group Members Participating physicians from the four intercontinental regions (*national coordinators): Hassan Salah*, Mohamed Abdelwahab, Mohamed Sayed, Sherif Khatab, Mervat El Gohary, Abdelrazeq Mohie Eldine, Ehab Alfons, Hassan Salah, Diaa Eldeen M. Abdel El Aal, Alaa Ismail, and Maged Elmohamedy (Egypt, Africa); Nadia Meziane*, Mohamed Aboutika, Nadia Maziane, Fatima Jamil, Larbi Rimi, Laila Zaouad, Abdelali Lasry, and Mohamed Outifa (Morocco, Africa); Godwin Olufemi Ajayi*, Christian Chigozie Makwe, Ian O Damole, Abidoye Gbadegesin, Ayodele Olantunji Arowojolu, Alaruru Olusoji Adeyanju, and Ephraim Okwudiri Ohazurike (Nigeria, Africa); Radhouane Rachdi*, Esseddine Sfar, Mohaed Faouzi Gara, Hedi Khiari, Bédis Mohamed Chennoufi, Hédi Rziga, Kais Chaabane, Mohamed Basli, Hdili Oueslati, and Leila Attia-Mahbouli (Tunisia, Africa); Leon C. Snyman*, Sagaren Naidu, Etienne Wilhelm Henn, Shayhana Ganesh, Sophie Mathijs, and Soma Priya Pillay (South Africa, Africa); Leyla Rzaguliyeva*, Zeynab Suleymanova, Sevinj Mammadova, Elmira Amirova Rzayeva, Malahat Abdullayeva, Gulnar Mammadli Mehdikhanli, Rana Huseynova, Sevil Rushani, Elmira Abdullayeva, Natig Maharramov, Irada Hajiyeva, Sevil Ramazanova Musayeva, Matanat Jahangirova, Sevinj Muradova Mammadova, and Nasiba Khudiyeva (Azerbaijan, Eurasia); Tatia Tugushi*, Nana Janelidze, Nato Khonelidzem, Natela Mikadze, Lia Panchulidze, Maia Tsereteli, Maia Janelidze, Pikria Kamladze, Khatuna Kereselidze, Bela Metreveli, Ketevan Osidze, Tamar Ubilava, Gia Tsagareishvili, Mzia Chikvaidze, Nani Marsagishvili, and Ketevan Shanidze (Georgia, Eurasia); Alexey Viktorovich Pyregov*, Marina Vladimirovna Duhanina, Galina Nikovaevna Parkanova, Sergey Vladimirovich Kirov, Tatiana Alekseevna Zhigulina, Irina Oseevna Turyan, Guzel Ravzalevna Husaenovna, Vladimir Nikolaevich Nikanorov, Olga Sergeevna Lavrenova, and Tatiana Urievna Boldyreva (Russian Federation, Eurasia); Dilbar Najmutdinova*, Botir Meiliev, Mavluda Khayrutdinova, Toshpulat Abdukarimov, Iosif Djatdaev, Guzal Niyazmatova, Khilola Mansurova, Nuritdin Shodmanov, Maria Baratova, Nurlan Akhmedov, and Elmira Azimova (Uzbekistan, Eurasia); Rowshan Begum*, Nigar Sultana, Meherun Nesa, Momtaj Begum, Halida Yasmin, Moriom Faruqi Shati, Nurun Nipa, Gul Anar, Begum Ara, Hasina Akhter, Utpala Mazumder, Fowzia Yasmin, Jesmin Akter, Kazi Begum, and Zinnat Nasreen (Bangladesh, South Asia); Vaishali Chavhan, Aswath Kumar, Mala Srivastava, Dilip Rane, Vandana Khanijo, Nitin Raithatha, Gita Khanna, Uma Devi, Manas Kundu, Kusum Zaveri, Kumud Pasricha, Rakhee Sahoo, Sonali Sardeshpande, Vandana Shetty, Cherry Shah, Rohit Gugutia, Indranil Saha, and Bhaskar Pal (India, South Asia); Nazli Hussain*, Rubeena Salahuddin, Naheed Bano, Tallat Iftikhar, Tahira Batool, Amina Salahuddin, Iffat Naheed, Nuzhat Rasheed, Pushpa Srichand, Nazli Hossain, and Safia Sultana (Pakistan, South Asia); Muna Abdulrazzaq Tahlak*, Mushin Iklas, Stuti Mishra, Wajiha Ajmal, Kasturi Mumigati, Babita Shetty, Khalid Al Kutish, Kolshan Mohamed, Kawthar Mansoor, and Sejal Devendra Surti (United Arab Emirates, Middle East); Mohammed Ghani Chabouk*, Iman Hussain, Ali Jawad Mufedah, Al Dahan Shighaf, Alwakeel Ahlam, Muhsin Thikraa, Abdul Razaq Wasnaa, Iman Hasan Flayeh, Jwad Almosawi Bushra, and Mohammed Ali Jawad Ulfat Alnakkash (Iraq, Middle East); Mazen Zibdeh*, Narges Faleh, Al Omari Omar, Raed Khalifa, Abeer Ennab, Ihsan Mbaideen, Sulaiman Thbait, Lama Al Fares, Mo'een Fida, Mustafa Al Sabbagh (Jordan, Middle East); Waleed Al Jassar*, Nahid Al Ali, Fatma Al Amiri, Hanan Al Salem (Kuwait, Middle East); Joe Abboud*, Richard Kharrat, Abed El Nasser Abou Khalil, Naji Afif, Georges Abi Tayeh, Joseph Bou Chedid, Patrick Zaarour, Walid Abou Rjeily (Lebanon, Middle East); Hassan Abduljabbar*, Ahmed Harby, Mona Nabeel, Alaa Zain-Eldein, Jihan Alhazmi, Sami Alansari (Saudia Arabia, Middle East).
