# Supplementary material for: Risk Assessment and Management of Venous Thromboembolism in Women during Pregnancy and Puerperium (SAVE): An International, Cross-sectional Study
Source: TH Open. 2018 Apr 4;2(2):e116–30. doi: 10.1055/s-0038-1635573 (PMC6524867; doi:10.1055/s-0038-1635573)
Supplement: Supplementary file 1 — Supplementary Material [file 10-1055-s-0038-1635573-s170027.pdf]

Supplementary Material

Reminder of Awareness of Risk Factors for Venous Thromboembolic (VTE) Disease during Pregnancy Diffused to Investigators before Starting the Study

1. **VTE risk assessment in pregnancy** takes into account intrinsic maternal risk factors, pregnancy-specific risk factors, and intercurrent risk factors. Risk factors are submitted to variations during pregnancy and *postpartum*: the global risk must therefore be evaluated dynamically.
2. ***To give examples:***
   (a) *Intrinsic maternal risk factors*: personal history of VTE, family history of VTE, known thrombophilia, age, high body mass index, ethnicity, smoking, diabetes mellitus, varicose veins, thrombophilia, etc.
   (b) *Pregnancy-specific risk factors*: assisted reproductive technology, hormone replacement treatment, placenta-mediated pregnancy complications (preeclampsia, intrauterine growth restriction, placental abruption, and stillbirth), delivery through caesarean section, obstetric hemorrhage, etc.
   (c) *Intercurrent risk factors*: trauma, surgery, infectious disease, outbreak of inflammatory disease, immobilization, etc.
3. **Risk factors for antenatal and postnatal VTE *differ*** (Jacobsen AF et al, J Thromb Haemost 2008; 6:905–912; Sultan AA et al, Blood 2013; 121: 3953–3961).
   (a) *Jacobsen AF*:
   ◦ Antenatal VTE:
   - BMI/immobilization.
     - 25 kg/m^2, no immobilization: $\times 2$.
     - $<$ 25 kg/m^2, immobilization: $\times 8$.
     - 25 kg/m^2, immobilization: $\times 62$.
   - Low weight gain $<$ 7 kg: $\times 2$.
   - ART.
     - Singleton: $\times 4$.
     - Twin: $\times 7$.
   - Spontaneous twins: $\times 3$.
   - Smoking 10–30 cigarette/day: $\times 2$.

   ◦ Postnatal VTE:
   - BMI/antenatal immobilization:
     - 25 kg/m^2, no immobilization: $\times 3$.
     - $<$ 25 kg/m^2, immobilization: $\times 11$.
     - 25 kg/m^2, immobilization: $\times 40$.
   - Placental ischemic disease:
     - Preeclampsia: $\times 3$.
     - Intrauterine growth restriction (IUGR): $\times 4$.
     - Preeclampsia and IUGR: $\times 6$.
   - Mode of delivery:
     - Acute caesarean, no infection: $\times 3$.
     - Any caesarean, infection: $\times 6$.
     - Vaginal delivery, infection: $\times 20$.
   - Per/postpartum bleeding:
     - $>$1,000 mL: $\times 4$.
     - $>$1,000 mL and surgery: $\times 12$.

   (b) *Sultan AA (adjusted relative increase of risks)*:
   ◦ *Antepartum* VTE:
   - Medical comorbidities:
     - Varicose veins: +120%.
     - Inflammatory bowel disease: +250%.
     - Urinary tract infection: +80%.
     - Preexisting diabetes mellitus: + 250%.

- ◦ *Postpartum* VTE:
  - – Medical comorbidities:
    - Varicose veins: +290%.
    - Inflammatory bowel disease: +300%.
    - Cardiac disease: +430%.
  - – BMI > 30 kg/m^2: +245%.
  - – Pregnancy complications:
    - Stillbirth: +300%.
    - Obstetric hemorrhage: + 150%.
    - Preterm birth: +130%.
    - Caesarean delivery: +90%.

4. **Values of average odds ratios for some risk factors for VTE during pregnancy in the literature**: (bold case: major risk factors, OR ≥ 6)

**Intrinsic maternal risk factors**	
Previous VTE	25
Age > 35 years	1.2–1.4
Weight: obesity	2.7
BMI ≥ 25	1.7–2.4
BMI > 30	4.4–5.3
Weight gain in pregnancy > 21 kg	1.6
Smoking	2.1
Current smoker	2.7
10–30 cigarette/day	3.4
Varicose veins	2.4
Heart disease	**3.2–7.1**
Sickle cell disease	**2.5–6.7**
Systemic lupus erythematosus	**2.3–8.7**
**Intercurrent risk factors**	
Anemia	1.6–2.6
Immobility	**7.7–10.8**
**Pregnancy-specific risk factors**	
Parity	
2	1.5
≥ 3	2.4
Multiple pregnancy	1.7–4.2
Twins	1.8–2.6
ART	1.8–4.3
Hyperemesis	2.5–4.4
Preeclampsia	2.9–3.1
Preeclampsia plus fetal growth restriction	**5.8**
Stillbirth	**6.3**
Preterm delivery < 37 weeks	2.4–2.7
Antepartum hemorrhage	2.3
Caesarean section	1.8–3.6
Emergency caesarean section	2.7
Caesarean section + postpartum infection	**6.2**
Obstetric hemorrhage	**9**
Postpartum hemorrhage (PPH)	1.2–1.3
PPH > 1 L	4.1
PPH + surgery	**12**
Transfusion	**3.6–7.6**
Postpartum infection	**4.1–6.1**

5. Globally:
   *Most of the identified risk factors induce a very limited increase in absolute risks.
   *Pharmacologic prophylaxis: in patients cumulating minor risk factors for VTE; or with a major risk factor.
   ***Postpartum: prophylaxis for an absolute risk value reaching 3% (OR: 6) (ACCP 2012).**

Remind of ACCP 2012:

Major risk factors (OR: 6): presence of at least one risk factor
suggests a risk of postpartum VTE = 3%
Immobility (strict bed rest for > 1 week in the antepartum period)
Postpartum hemorrhage > 1,000 mL with surgery
Previous VTE
Preeclampsia with fetal growth restriction
Thrombophilia
Antithrombin deficiency
Factor V Leiden (homozygous or heterozygous)
Prothrombin G20210A (homozygous or heterozygous)
Medical conditions
Systemic lupus erythematosus
Heart disease
Sickle cell disease
Blood transfusion
Postpartum infection
Minor risk factors (OR >6 when combined): presence of at least two risk factors or one risk factor in the setting of emergency
Caesarean section suggests a risk of postpartum VTE > 3%
BMI > 30 kg/m^2
Multiple pregnancy
Postpartum hemorrhage > 1 L
Smoking > 10 cigarette/day
Fetal growth restriction (gestational age + sex-adjusted birth weight < 25th percentile)
Thrombophilia
Protein C deficiency
Protein S deficiency
Preeclampsia

*Antepartum:

Which absolute risk threshold value is legitimating a pharmacologic prophylaxis? Not easy, long and costly treatments, no current consensus; from 3 to 10%.

ACCP 2012 Summary of Guideline for Thromboprophylaxis in Pregnant Women

Caesarean Section

6.2.1. For women undergoing caesarean section without additional thrombosis risk factors, we recommend against the use of thrombosis prophylaxis other than early mobilization (Grade 1B).
6.2.2. For women at increased risk of VTE after caesarean section because of the presence of one major or at least two minor risk factors, we suggest pharmacologic thromboprophylaxis (prophylactic low-molecular-weight heparin [LMWH]), or mechanical prophylaxis (elastic stockings or intermittent pneumatic compression) in those with contraindications to anticoagulants while in the hospital following delivery rather than no prophylaxis (Grade 2B).
6.2.3. For women undergoing caesarean section who are considered to be at very high risk for VTE and who have multiple additional risk factors for thromboembolism that persist in the puerperium, we suggest that prophylactic LMWH be combined with elastic stockings and/or intermittent pneumatic compression over LMWH alone (Grade 2C).
6.2.4. For selected high-risk patients in whom significant risk factors persist following delivery, we suggest extended prophylaxis (up to 6 weeks after delivery) following discharge from the hospital (Grade 2C).

Previous VTE

8.2.1. For all pregnant women with prior VTE, we suggest postpartum prophylaxis for 6 weeks with prophylactic- or intermediate-dose LMWH or vitamin K antagonists targeted at INR 2.0 to 3.0 rather than no prophylaxis (Grade 2B).
8.2.2. For pregnant women at low risk of recurrent VTE (single episode of VTE associated with a transient risk factor not related to pregnancy or use of estrogen), we suggest clinical vigilance antepartum rather than antepartum prophylaxis (Grade 2C).
8.2.3. For pregnant women at moderate to high risk of recurrent VTE (single unprovoked VTE, pregnancy- or estrogen-related VTE, or multiple prior unprovoked VTE not receiving long-term anticoagulation), we suggest antepartum prophylaxis with prophylactic- or intermediate-dose LMWH rather than clinical vigilance or routine care (Grade 2C).
8.2.4. For pregnant women receiving long-term vitamin K antagonists, we suggest adjusted-dose LMWH or 75% of a therapeutic dose of LMWH throughout pregnancy followed by resumption of long-term anticoagulants postpartum rather than prophylactic-dose LMWH (Grade 2C).

Thrombophilia

9.2.1. For pregnant women with no prior history of VTE who are known to be homozygous for factor V Leiden or the prothrombin 20210A mutation and have a positive family history for VTE, we suggest antepartum prophylaxis with prophylactic- or intermediate-dose LMWH and postpartum prophylaxis for 6 weeks with prophylactic- or intermediate-dose LMWH or vitamin K antagonists targeted at INR 2.0 to 3.0 rather than no prophylaxis (Grade 2B).
9.2.2. For pregnant women with all other thrombophilias and no prior VTE who have a positive family history for VTE, we suggest antepartum clinical vigilance and postpartum prophylaxis with prophylactic- or intermediate-dose LMWH or, in women who are not protein C or S deficient, vitamin K antagonists targeted at INR 2.0 to 3.0 rather than routine care (Grade 2C).
9.2.3. For pregnant women with no prior history of VTE who are known to be homozygous for factor V Leiden or the prothrombin 20210A mutation and who do not have a positive family history for VTE, we suggest antepartum clinical vigilance and postpartum prophylaxis for 6 weeks with prophylactic- or intermediate-dose LMWH or vitamin K antagonists targeted at INR 2.0 to 3.0 rather than routine care (Grade 2B).
9.2.4. For pregnant women with all other thrombophilias and no prior VTE who do not have a positive family history for VTE, we suggest antepartum and postpartum clinical vigilance rather than pharmacologic prophylaxis (Grade 2C).
6.2.4. For selected high-risk patients in whom significant risk factors persist following delivery, we suggest extended prophylaxis (up to 6 weeks after delivery) following discharge from the hospital (Grade 2C).

Remind of RCOG 2015: Risk Assessment for Venous Thromboembolism

- **If total score ≥ 4 antenatally, consider thromboprophylaxis from the first trimester.**
- **If total score 3 antenatally, consider thromboprophylaxis from 28 weeks.**
- **If total score ≥ 2 postnatally, consider thromboprophylaxis for at least 10 days.**
- **If admitted to hospital antenatally, consider thromboprophylaxis.**
- **If prolonged admission (≥ 3 days) or readmission to hospital within the puerperium, consider thromboprophylaxis.**

For patients with an identified bleeding risk, the balance of risks of bleeding and thrombosis should be discussed in consultation with a hematologist with expertise in thrombosis and bleeding in pregnancy.

**Risk factors for VTE**	
Preexisting risk factors	Score
Previous VTE (except a single event related to major surgery)	4
Previous VTE provoked by major surgery	3
Known high-risk thrombophilia	3
Medical comorbidities, e.g., cancer, heart failure; active systemic lupus erythematosus, inflammatory polyarthropathy or inflammatory bowel disease; nephrotic syndrome; type I diabetes mellitus with nephropathy; sickle cell disease; current intravenous drug user	3
Family history of unprovoked or estrogen-related VTE in first-degree relative	1
Known low-risk thrombophilia (no VTE)	1a
Age (> 35 years)	1
Obesity	1 or 2
Parity ≥ 3	1
Smoker	1
Gross varicose veins	1
Obstetric risk factors	
Preeclampsia in current pregnancy	1
ART/IVF (antenatal only)	1
Multiple pregnancy	1
Caesarean section in labor	2
Elective caesarean section	1
Mid-cavity or rotational operative delivery	1
Prolonged labor (> 24 hours)	1
PPH (> 1 L or transfusion)	1
Preterm birth < 37 + 0 weeks in current pregnancy	1
Stillbirth in current pregnancy	1
Transient risk factors	
Any surgical procedure in pregnancy or puerperium except immediate repair of the perineum, e.g., appendicectomy, postpartum sterilization	3
Hyperemesis	3
OHSS (first trimester only)	4
Current systemic infection	1
Immobility, dehydration	1
**TOTAL:**	

RCOG 2015 Summary of Guideline for Thromboprophylaxis in Women with Previous VTE and/or Thrombophilia

Very high risk	Previous VTE on long-term oral anticoagulant therapy<br>Antithrombin deficiency Antiphospholipid syndrome with previous VTE	Recommend antenatal high-dose LMWH and at least 6 weeks' postnatal LMWH or until switched back to oral anticoagulant therapy<br>*These women require specialist management by experts in hemostasis and pregnancy*
High risk	Any previous VTE (except a single VTE related to major surgery)	Recommend antenatal and 6 weeks' postnatal prophylactic LMWH
Intermediate risk	Asymptomatic high-risk thrombophilia homozygous factor V Leiden/compound heterozygote protein C or S deficiency<br>Single previous VTE associated with major surgery without thrombophilia, family history or other risk factors	Refer to local expert<br>Consider antenatal LMWH<br>Recommend postnatal prophylactic LMWH for 6 weeks<br>Consider antenatal LMWH (but not routinely recommended)<br>Recommend LMWH from 28 weeks of gestation and 6 weeks' postnatal prophylactic LMWH
Low risk	Asymptomatic low-risk thrombophilia (prothrombin gene mutation or factor V Leiden)	Consider as a risk factor and score appropriately<br>Recommend 10 days' postnatal prophylactic LMWH if other risk factor postpartum (or 6 weeks' if significant family history)
